# Challenges perceived by social workers to prevent FGM/C in Bavaria: a prospective, cross-sectional survey

**DOI:** 10.1186/s12905-024-03154-4

**Published:** 2024-06-14

**Authors:** N. Seifert, E. Mürdter, NC. Schmidt

**Affiliations:** 1 DigiHealth Institute, Neu-Ulm University of Applied Science, Neu-Ulm, Germany; 2 Faculty of Social Science, Katholische Stiftungshochschule München/ Catholic University of Applied Science, Preysingstrasse 95, 81667 Munich, Germany; 3 Gynaecology Division, Department of Paediatrics, Gynaecology and Obstetrics, University Hospitals of Geneva, Geneva, Switzerland

**Keywords:** Female genital mutilation/cutting, FGM/C, Prevention, Social work, Training

## Abstract

**Background:**

Worldwide, at least 230 million girls and women are affected by female genital mutilation/ cutting (FGM/C). FGM/C violates human rights and can cause irreparable harm and even lead to death. In 2022, more than 100,000 survivors of FGM/C lived in Germany, and more than 17,000 girls were considered at risk. Due to the increasing number, there is a need to improve the skills of professionals not only to treat FGM/C but also to prevent it, aiming to maintain or improve women’s physical and mental health. However, previous studies mostly focused on health care providers, even though other professionals such as social workers, play an important role in the provision of sexual and reproductive health (SRH) care and are often the first point of contact. Therefore, the study’s main objective was to understand challenges perceived by social workers in pregnancy counselling centres in the provision of good quality of SRH care for girls and women suffering from or endangered by FGM/C.

**Methods:**

A quantitative self-administered cross-sectional online survey was sent by e-mail in 2021 to all pregnancy counselling centers in the German federal state of Bavaria.

**Results:**

Among the 141 participants, 82% reported no or insufficient FGM/C knowledge and barriers to provide the best quality of care. The main findings were language obstacles (82.7%), perceived client’s fear or shame (67.9%) and cultural difficulties (45.7%). Furthermore, participants also reported a lack of competence on the professional side (29.6%). Importantly, most participants (129 of 141; 92%) expressed interest in training.

**Conclusion:**

Providing comprehensive good quality sexual and reproductive health care to women and girls affected from or endangered by FGM/C is challenging. The study revealed the importance of strengthening the skills of social workers and suggested the following strategies: (1) enhancing FGM/C knowledge and skills (including specialized competences e.g., in mental health) by improving training and information material for the target group, (2) improving referral pathways and addressing deficits in the existing care system (e.g. with health or legal institutions), and (3) developing trusting relationships with cultural (or traditional) mediators to build strong community networks.

## Introduction / background

The World Health Organisation (WHO) defines female genital mutilation as “all procedures that involve the partial or total removal of external genitalia or other injury to the female genital organs for non-medical reasons” [1] Female genital mutilation (FGM) violates human rights and is condemned in international treaties and conventions and by national laws in many countries [2].It has been recognized as a form of discrimination against women and can cause irreparable harm to girls and women and even lead to death [Box 1] [1, 4].

**Box. 1:** Immediate or long-term complications of FGM (own representation based on [1, 3]

**Immediate complications**	
(severe) pain. infections. (severe) bleeding. infections. injury to adjacent organs (e.g. abscess formation. fractures. septic shock or death)	
**Long-term complications**	
Increased risk of urinary problems (e.g. dysuria or urinary tract infections). vaginal problems. menstrual problems (e.g. painful menstruation) or sexual problems (pain during intercourse etc.)	
Birth complications such as prolonged labor. difficult delivery. perineal tears or excessive bleeding	
Psychological disorders or trauma	

Worldwide, at least 230 million girls and women are affected by female genital mutilation/ cutting (FGM/C) [5], classified according to the WHO in four types [Table 1]. According to representative surveys, FGM/C is practised in at least 30 countries in Africa, the Middle East and Asia. Reasons for the practice of FGM/C vary between countries but also among different ethnicities within a country. The main four reasons include psychosexual arguments (such as the control of women’s sexuality), sociological and cultural rites (it might be seen for example as a requirement for marriage), hygiene and aesthetic reasons but also religious justifications even though neither the Islam nor Christianity endorse FGM/C) [7]. 

**Table 1:** Classification of female genital mutilation according to the World Health Organization (Courtesy of [6])

Type I: Partial or total removal of the clitoris* and/or the prepuce (clitoridectomy)
Type Ia: removal of clitoral hood or prepuce only
Type Ib: removal of clitoris* with prepuce
Type II: Partial or total removal of the clitoris* and labia minora. with/without excision of labia majora (excision)
Type IIa: removal of labia minora only
Type IIb: partial or total removal of clitoris* and labia minora
Type IIc: partial or total removal of clitoris.* labia minora. and labia majora
**Type III: Narrowing of the vaginal orifice with creation of a covering seal by cutting and apposition the labia minora and/or the labia majora. with or without excision of the clitoris (infibulation)**
Type IIIa: removal and apposition of labia minora
Type IIIb: removal and apposition of labia majora
**Type IV: unclassified**
All other harmful procedures to the female genitalia for non-medical purposes (e.g. pricking. piercing. incising. scraping. cauterization)

*Removal of the clitoris corresponds to removal of the glans of the clitoris. **not** the entire organ

In respect to the prevalence, important variations exist in respect to FGM/C prevalence rates between countries but regional inside countries; for example, data from Somalia or Eritrea, report that more than 80% of women and girls between 15 and 49 years of age are affected, while in countries such as Cameroon or Uganda, the prevalence rate is approximately 1% [2]. However, estimating the total number of girls and women affected by FGM/C remains a challenge due to difficulties in data collection and, therefore, the difficulty for cross-country comparisons [8].

In Germany, national data on FGM/C prevalence derive from indirect estimates. This means that recent FGM/C prevalence data from UNICEF are multiplied with the absolute, most current numbers of female permanent residents (in the first generation) having moved to Germany from 31 countries where FGM/C is practiced. For girls at risk of FGM/C (first or second-generation migrants) data are calculated based on the recommendations of the European Institute of Gender Equality (EIGE) suggesting a high-risk vs. low-risk scenario (assuming no effect of migration or acculturation on FGM/C vs. reducing the FGM/C risk)^1^. The number of women and girls living in Germany with a nationality from one of the countries where FGM/C is practised has risen by nearly 40% since 2017. According to different estimates, in 2022 almost 103,947 women were living in Germany who had experienced FGM/C prior to their arrival in the country [9, 10]. Furthermore, more than 17,721 girls were considered at risk [9, 10]. The six main nationalities of these women and girls living in Germany were Nigerian, Eritrean, Ghanaian, Indonesian, Somalian and Iraqi [9, 10]. Given the increase of women and girls either living with FGM/C or endangered by FGM/C in Germany, there is a need to improve the skills of service providers not only to treat FGM/C but also to prevent it [11]. However, previous studies reported that knowledge about FGM/C among professionals is insufficient and highlighted the need for further training [3, 11, 12]. But most of these studies focused on health care providers only. Few studies have included other professionals such as social workers, even though they are in contact with clients in various relevant areas, such as child welfare, law related areas, or advocacy. As illustrated by Costello social and welfare workers have a responsibility “to advocate the rights of girls not to be cut” and legal actions can be required to protect the girl [4]. Furthermore, studies also describe that social or welfare workers judgment can be challenged due to the fear to stigmatize clients. Therefore, Patrick and Markiewicz highlight in an Australian study the importance to develop frameworks for those working in cross-cultural settings [13].

Also, social workers in Germany are an important part of the healthcare system. They are working not only in the clinical sector but are also supporting the school entry examinations or are working in pregnancy counselling centres. Pregnancy counselling centres are financed by the German government and answer free of charge to different needs of women and their families, mainly during pregnancy and in the first three years after childbirth. The primary services include (i) counselling, social and economic assistance during this period (ii) advice and counselling in case of ambivalence during pregnancy or the wish to terminate a pregnancy and (iii) family planning education, but also support and advice in case of an unfulfilled fertility desire. These services are provided free of charge to all women and families in Germany, but some services such as social and economic assistance around childbirth are often utilized by vulnerable populations (e.g. migrant women and/or very young pregnant women). Social workers are especially trained for those offered services and are the main professionals working in pregnancy counselling centres.

In this context, social and health care workers in pregnancy counselling centres are one of the key groups working with clients from FGM/C countries. Often, they find themselves confronted with questions targeting education around FGM/C prevention or existing laws (e.g. section 226a of the Criminal Code stating that under German law performing FGM/C is a criminal offense and can be sentences with up to 15 years in prison). Also, social workers, who are working in pregnancy counselling centers are recognized by the state and are obliged (such as other registered health and social care professionals) to provide protection if there is evidence of a FGM/C endangerment. (§ 4 (2) German Act on Cooperation and Information in Child Protection (KKG)).

Other questions include seeking advice regarding support services in Germany, such as medical services experienced with FGM/C during childbirth or in need of legal assistance during the asylum-seeking process. In consequence, health professionals and social workers need sufficient information about FGM/C and its impact to address this issue sensitively, aiming to prevent further harm and address fear, experience of racism and exclusion at the same time [7].

Therefore, the study’s main objective was to understand challenges perceived by social workers and other health professionals working in pregnancy counselling centres in the provision of good quality sexual and reproductive health care for girls and women suffering or endangered by FGM/C. The study also aimed to evaluate referral pathways for women with FGM/C or girls at risk for FGM/C. A secondary objective of the study was to understand the training needs of social workers and health professionals in pregnancy counselling centres in Bavaria, Germany.

## Materials and methods

### Study design

This quantitative cross-sectional survey was conducted in response to the FGM/C prevention strategy of the Bavarian State Ministry for Family Affairs, Labour, and Social Affairs, which was launched in 2020 to address FGM/C violence against women and girls living in the state. The online questionnaire addressing professionals in pregnancy counselling centres in Bavaria was developed as a survey instrument based on previous research of literature of health professionals’ knowledge, attitudes, training, and clinical practice [12, 14] and a prior work of the study team about FGM/C knowledge and training needs among health care workers in Freiburg [3]. Inclusion criteria included all professionals aged over 18 years working in pregnancy counselling centres and/ or specialised FGM/C advice centres^2^ in Bavaria and agreement to the informed consent form before starting the online questionnaire.

The questionnaire contained both open (free text format) and closed questions (single or multiple choice, including Likert scales), across the following four categories:

(i) socio-demographic factors.
(ii) self-estimated knowledge of FGM/C.
(iii) challenges in referral pathways.
(iv) training needs.

The study received approval (No. 2021/N15) from the ethical review board of the Catholic University of Applied Science, Munich, in May 2021.

### Data collection

The online survey was conducted from 15th June to 16th July 2021 using the SoSci Survey tool. The online questionnaire was offered in German only, as German language skills are a prerequisite to work in a pregnancy counselling centre. An invitation to participate, including a link to the online survey, was sent via an official e-mail distribution list to all 144 pregnancy counselling centres, complemented by a reminder two weeks before closure. According to the data of the online tool, respondents needed 10–15 min to answer the questionnaire.

### Data preparation and analysis

In total, 232 participants started the survey, but 79 participants stopped answering the questionnaire after the first or second question. Data were checked for plausibility before evaluation, and incomplete questionnaires (missing answers over 10%) or those that did not meet the inclusion criteria (one questionnaire was answered from outside Bavaria) were excluded.

Thus, a final data set of 141 valid cases (61%) could be analysed. Some questions, especially in the third category (evaluating referral pathways), were only for participants possessing FGM-knowledge or experience. Therefore, the absolute sample size ‘n’ used for the percentage calculation varies for some questions.

The evaluation was carried out descriptively using frequencies and percentages. Chi-square independence test, bivariate correlation according to Spearman, and correlation according to Phi were also carried out with a significance level of α = 0.05. The answers given in free texts were summarized by content analysis and transferred into a category system. All descriptive statistical analysis was done using IBM SPSS version 27.

## Results

### Participant data

As shown in Table 2, the study participants were predominantly female (98.6%), worked in pregnancy counselling centres (92%) and about half of the respondents (51.8%) were between 51 and 65 years old. 97.2% lived in Germany since birth, and most possessed a degree in social work (87.9%). Participants came from all nine administrative districts in Bavaria, and almost half of the participants (41.8%) worked in cities with 20,001-100,000 inhabitants.

**Table 2:** Characteristics of the participants

Variable	Frequency (*n*)	Percentage (%)
Total	141	100
**Gender**		
Female	139	98.6
Male	2	1.4
**Age**		
18–30 years	13	9.2
31–50 years	55	39.0
51–65 years	73	51.8
**Period of residence in Germany**		
Since birth	137	97.2
More than 10 years	4	2.8
**Government district**		
Upper Bavaria	52	36.9
Central Franconia	20	14.2
Lower Franconia	18	12.8
Lower Bavaria	17	12.0
Swabia	16	11.3
Upper Palatinate	10	7.1
Upper Franconia	8	5.7
**Population density of the workplace**		
5.001–20.000 Inhabitants	26	18.4
20.001–100.000 Inhabitants	59	41.9
100.001–500.000 Inhabitants	37	26.2
> 500.000 Inhabitants	19	13.5
**Workplace**		
State-recognised counselling centres for pregnancy issues	114	80.9
Catholic Counselling Centre for Pregnancy Issues	10	7.1
(Specialist) centres for pregnancy issues	5	3.5
Other specialised counselling centres (Human trafficking, forced marriage. refugee and integration counselling)	5	3.5
Other areas	7	5.0
**Highest level of education**		
Social pedagogical studies	124	88.0
Other studies	11	7.8
College of higher education	4	2.8
Other educational qualification	2	1.4

### FGM/C knowledge and contacts

82% of the total participants (*n* = 141) self-reported none or insufficient FGM/C knowledge, with only 18% self-evaluating their knowledge as good or excellent. However, when participants with any form of FGM/C knowledge (optimizable, good, or excellent) were asked additional questions in respect to the prevalence of FGM/C in Germany, the different forms of FGM/C according to the WHO classification and the existence of a law against FGM/C in Germany, knowledge deficits were revealed: less than half knew the estimates of girls or women affected by FGM/C in Germany, and only 36% knew how many forms of FGM/C exist according to the WHO classification [Table 3].

**Table 3:** FGM-Knowledge

Variable	Frequency (*n*)	Percentage
**Self-estimated FGM-Knowledge** (*n* = 141)		
• good to excellent knowledge	25	17.7
• optimizable	100	70.9
• no closer knowledge	16	11.4
**Estimated number of girls and women affected by FGM in Germany** (*n* = 125)		
0–20.000 affected	3	2.4
20.001–40.000 affected	13	10.4
40.001–60.000 affected	19	15.2
60.001–80.000 affected	52	41.6
80.001–100.000 affected	5	4.0
More than 100.000 affected	6	4.8
I unfortunately do not know	27	21.6
**Known FGM forms according to WHO** (*n* = 125)		
I	0	0
II	3	2.4
III	35	28.0
IV	46	36.8
V	4	3.2
I unfortunately do not know	37	29.6
**Law against FGM** (*n* = 125)		
Yes	94	75.2
No	7	5.6
I unfortunately do not know	24	19.2

Among the 141 participants, more than half (57%, *n* = 81) worked with girls and women threatened or affected by FGM/C. Of these, most reported (62%) in between one up to ten contacts per year, while 17% reporting more than 20 contacts per year. Increasing numbers of working connections with those affected/threatened correlated with higher knowledge about FGM/C (correlation coefficient ρ = 0.227, *p* = 0.042). Also, the number of contacts with affected or threatened girls or women correlated with increased population density of the working area (correlation coefficient ρ = 0.329, *p* = 0.003).

### Perceived challenges during counselling provision

The most critical challenges in contact with females affected by FGM/C were language barriers (82.7%) and around a quarter reported a lack of funding for interpreters (28.4%) and limited access to cultural mediators (22.4%). Furthermore, mentioned challenges in counselling due to perceived client’s fear or shame (67.9%), impression of cultural barriers (45.7%), but also provider’s self-perceived lack of competence to address FGM/C (29.6%) or missing medical knowledge (16.1%).

### Addressing FGM

Professionals (*n* = 81) having contact with by FGM/C endangered or affected females reported that the topic of FGM/C was rarely (53%) or never (42%) raised by the clients themselves during counselling. Also, only 3.7% of the social workers mentioned FGM/ C during a general pregnancy counselling situation e.g. in case of requests for financial assistance. The majority (75.3%) addressed the topic only if it seemed relevant for the contact with the client e.g. in case of questions in respect to asylum requests or search for a gynaecologist. Notably, 17.3% highlighted that they did not know how to address the issue, and 3.7% had never thought about it.

### Perception of FGM/C referral pathways

**Fig. 1:**
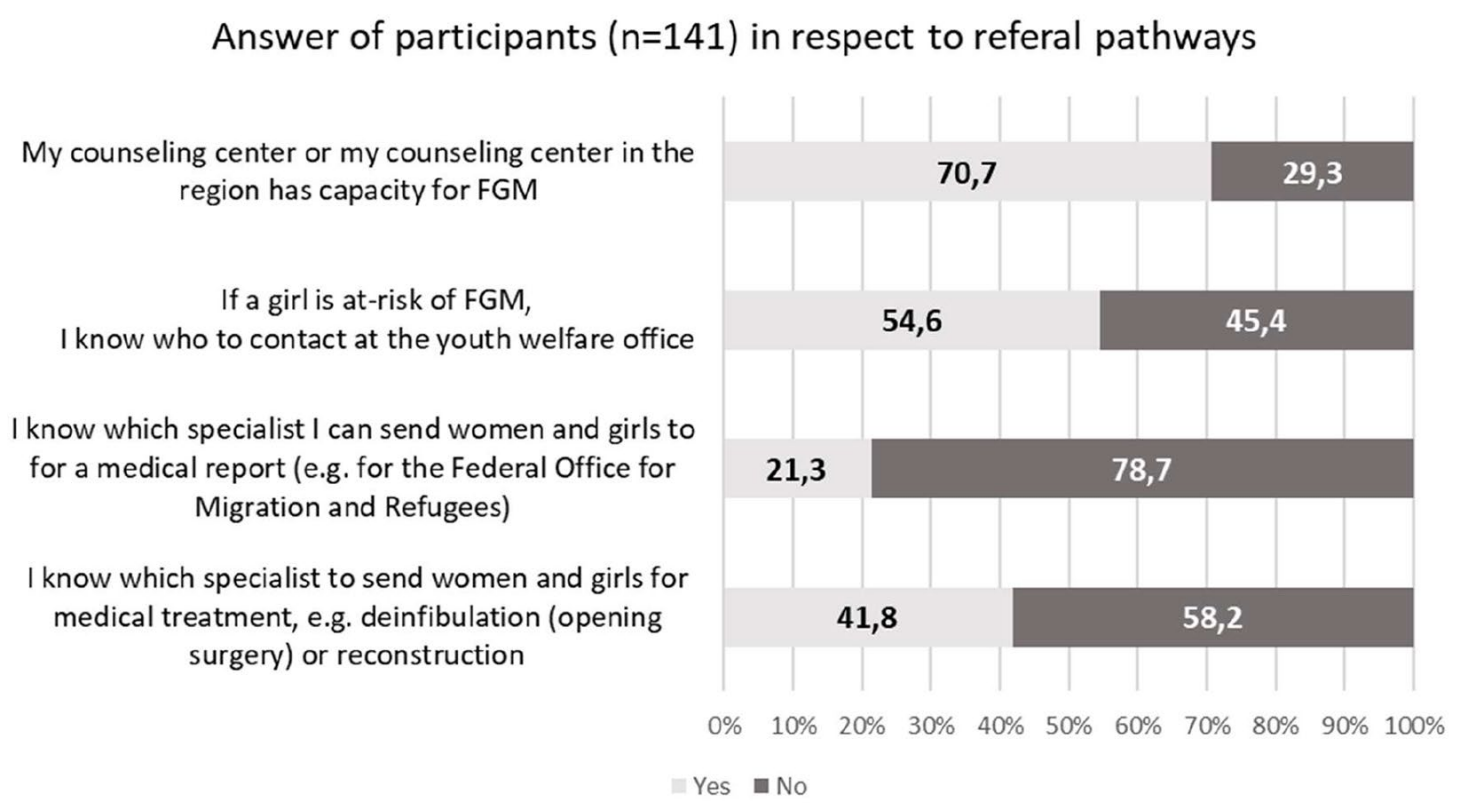
Answer of participants (in %) in respect to referral pathways

Caring for those affected or threatened by FGM/C requires a multidisciplinary approach, including other professionals from the health and social sector as well as community support or legal expertise. Half (62.4%) of the social workers in our study reported that social workers at their counselling centre did not know enough about FGM. Furthermore, more than three-quarters of social workers (79%) were unsure if medical experts for FGM/C survivors existed in their region. This lack of information was especially noted regarding experts writing medical reports and providing medical treatment such as, defibulation (58%) [Fig. 1]. Similar deficits were mentioned in respect to experts providing legal counselling for women and girls at risk: more than half of respondents perceived the provision of legal counselling as insufficient (60.3%) and 21% were not aware about the existence of legal counselling for women and girls in their regions.

Furthermore, participants believed that few paediatricians or gynaecologists (6.4% vs. 5%) addressed FGM/C actively when the client did not mention it, even as it was perceived essential for child protection.

Two-quarters of participants reported that local FGM/C networks either did not exist in their regions or were unaware of it (45.4% vs. 33.3%), possibly leading to 45% of participants stating that no direct personal contacts existed with the local child protection services. Besides deficiencies in the referral pathways, the current care situation in Bavaria was judged as needing improvement especially in respect to the following aspects:

- In less than 20% of the regions, *support by cultural mediators* was perceived as guaranteed.
- Nearly 60% perceived *legal counselling* as insufficient, and 21% did not know the topic.
- More than 43% perceived the *availability of care provided by gynaecologists* as insufficient, while more than 38% did not know about it.
- Furthermore, participants were unsure if gynaecologists and midwives actively addressed FGM/C during pregnancy (67%) or whether paediatricians in the region were sensitized to FGM/C (62%).

### Information about FGM/C

To address the topic of FGM/C with clients almost half of the respondents (*n* = 141, 49.7%) only used oral explanations. Approximately a quarter (28.4%) worked with written brochures or flyers, and 14.9% used websites during the pregnancy counselling with clients. A small proportion already utilized films/podcasts (2.8%) or social media (1.4%). However, only 7.8% of participants judged the available information material as sufficient, while more than 31.9% found it insufficient, and 26.2% did even not possess any information material in the centers.

Improvement and expansion of current information materials on FGM/C are needed in all six regional areas. In this respect, participants mainly considered written brochures or flyers as most helpful (85.8%), followed by materials on the internet (61%), films (36.9%), podcasts (9.9%), or other materials (5.7%). Preferred languages for resources related to nationalities of clients, mainly from the African continent, but also English, French, or German.

### Training needs

32.6% of the participants have already attended a training course, while 67.4% of the social workers never attended training on FGM. Overall, the interest in further FGM/C training was very high (92%). Only 4% of participants were not interested, while 4% judged their knowledge as already sufficient.

Training areas included acute and chronic FGM/C symptoms in the field of general medicine, but also mental health or questions related to pregnancy (69.8%). Further identified training needs included skills to address FGM/C with women/girls and their relatives (52.7%); basic knowledge about FGM/C forms and causes (47.3%); but also, casework on challenging situations including counselling techniques (32.6%); the legal status in asylum procedures (24.8%) or child protection (14.7%). The choice of the desired training topics showed that participants without prior FGM/C education expressed an interest in basic FGM/C knowledge (χ2 = 18.95, df = 2, *p* = 0.00, Cramer V = 0.383) or the impact of FGM/C on somatic or psychosomatic health. (χ2 = 11.50, df = 2, *p* = 0.003, Cramer V = 0.299). On the other hand, those who had already attended FGM/C teaching sessions desired more training on dealing with challenging situations and improving counselling techniques (χ2 = 12.73, df = 2, *p* = 0.002, Cramer V = 0.314). At the same time, professionals with FGM/C expertise were particularly interested in improving community work (religious communities, role of men) (χ2 = 12.10, df = 2, *p* = 0.002, Cramer V = 0.306).

Regarding the form of training (*n* = 129), the majority favoured a combination of face-to-face training and digital methods (32.6%). Approximately a quarter expressed a preference for face-to-face only (28.7%) vs. only digital synchronous (23.3%) training. Only 10.9% desired digital asynchronous options (i.e., via video tutorials) that everyone can view at any time. In respect to the time frame of the training (*n* = 110), almost half (42.7%) of the social workers opted for full-day training, while 31.8% favoured individually bookable modules on the respective area of interest, 15.5% half-day training, and only 8.1% preferred two-hour trainings over an extended period of time.

## Discussion

This study is, to our knowledge, the first cross-sectional survey accessing FGM/C challenges in the provision to good quality of care perceived by social workers and professionals working at pregnancy counselling centres in Bavaria. Due to the increasing numbers of girls and women migrating from countries where FGM/C is practiced to European countries, health professionals and social workers are more likely to be in contact with women or girls affected or threatened by FGM. While a great need for training has been already recognized in the medical field, our study showed an important additional training need for social workers [3, 11, 12, 15]. In a recent study conducted by Molina-Gallego et al. [16] in Switzerland among 1168 health professionals, only 13.8% of participants had received FGM/C training. Importantly, in our study, 82% self-evaluated their knowledge as insufficient, and 92% of participants expressed desire in training. The lack of FGM/C knowledge and the insecurity of addressing the topic might partially explain why very few providers routinely addressed FGM/C in general pregnancy counselling situations, even if they are often the first contact for girls and women. As Käkelä and colleagues pointed out, a significant challenge in service provision is the cultural sensitivity to identify girls and women at risk or potentially affected and highlights the need to train social workers [17]. The importance for cross-cultural sensitive training including self-reflection has been also highlighted by Costello to support women’s and girl’s decision to discuss the topic but also to mediate intergenerational conflicts [4]. This should also include critical self-reflection how to approach FGM/C in respect to gender and race [18].

Thus, considering the need of women and girls in respect to their sexual and reproductive health and the essential interest on the side of social workers, institutions and stakeholders should reflect on how to implement FGM/C training. This includes basic FGM/C knowledge for those newly getting in touch with the topic, including skills on how to address FGM/C culturally adequate. Furthermore, training should not only respond to the needs of social workers with respect to the forms of training (including virtual forms and face-to-face training), but also regarding the provision of information resources as this was mainly perceived as insufficient. Developing or adapting training resources to the specific needs of professionals, as already done for gynaecology and obstetrics, is an important task to be completed in the future [17]. FGM/C training should not only be included in the academic education for social workers from a practical perspective but also be informed by the theoretical frameworks of social justice and human rights. Furthermore, qualitative approaches should be considered to elaborated on the challenges of professionals but also clients in pregnancy counselling centers.

However, as Abdulcadir and colleagues noted, little evidence exists on the impact of training efforts. Therefore, implementation of training, regardless of its form, should include measures to assess the outcomes of such interventions [11].

Another essential aspect mentioned by the participants was that training should include establishing referral pathways to provide comprehensive sexual and reproductive health care for women and girls. In a recent publication, Njue and colleagues noted that the lack of clear referral pathways for FGM/C in Australia endangers the continuum of care for women with FGM/C [19]. Our survey confirmed this finding because providers were either unaware of the availability of comprehensive services or evaluated them as insufficient or non-existent. This is especially important as clear referral pathways are crucial for women with FGM/C or endangered girls. However, until now, empirical evidence in German-speaking countries mainly focused on health professionals’ knowledge, either in medical doctors or midwives [3, 15]. Therefore, our study highlights a critical need to provide infrastructural support and suggests helpful instruments facilitating the process. Interdisciplinary work in between medical professionals and social workers at pregnancy counselling centres would allow to improve social, health and legal care for FGM/C survivors and those who are endangered by FGM/C. As Njue and colleagues (2021) suggested, referral algorithms or electronic patient records with ‘drop-down menus’ for referral sites for health complications of FGM/C could improve the care for women and girls. Future studies should therefore compare innovative instruments to improve referral pathways. Besides technical tools, it is crucial to involve community organizations to facilitate cooperation with parents, other family members, and community members [19–21]. But also, the involvement of cultural mediators can be perceived as a key factor in the provision of prevention and care. Cultural mediators are well recognized in their respective community and can be ambassadors, but they also convey technical information on FGM/C culturally sensitively: using norms, laws, and feelings, communicating, and promoting strategies and skills within a group or community [4, 12, 22, 23]. As nearly two-thirds of participants in our survey perceived the collaboration with cultural mediators as insufficient, there is an important call to involve culture mediators in programs.

### Strengths and limitations

Some limitations should be considered when interpreting the results. First, as the ministry of social affairs distributed the survey, a selection bias needs to be considered. Using the mailing list of the Bavarian Ministry of Social Affairs the questionnaire was sent mainly to the head of the pregnancy counselling centres. Assuming that they largely responded to the questionnaire themselves (prior to forwarding it), this might explain partly that over 50% of respondents were more experienced women in between 51 years to 65 years. Furthermore, due to the nature of self-reporting, a potential bias may occur. First of all, the Bavarian Ministry of Social Affairs allocates funding to the pregnancy counselling centers, thus a social desirability bias needs to be considered, potentially resulting in underestimating training needs or other reported challenges. However, respondents perceived important challenges that are comparable to results reported by health professionals in other studies. Therefore, a self-reporting bias on the study outcomes seems to be rather small. Furthermore, a recall bias might affect especially those participants working in pregnancy counselling centers with low numbers of women or girls affected or endangered by FGM/C. Therefore, future studies should evaluate the effect of recall bias in this kind of survey by including more participants. Lastly, dropout-attrition (after the first two questions) was higher than normally expected. As respondent fatigue is considered rather small after the first two questions, potentially timing and location of the questionnaire (during routine pregnancy counselling) can be seen as a major limitation which should be improved in future studies.

Despite these limitations, the present study has important strengths. First, by using the e-mail distribution list of the Bavarian Ministry of Social Affairs, we were able to give all pregnancy counselling centers in Bavaria the opportunity to participate. Second, 141 participants from all administrative districts in Bavaria answered the questionnaire and various demographic, knowledge, and experience related factors were analysed to create a comprehensive picture. Furthermore, we consider the results as representative for the target population as participants with similar characteristics (in respect to educational level and gender) from all administrative regions in Bavaria answered the survey. Additionally, as the sample size is sufficiently large without important outliers distorting the picture and data are comparable to international studies, we consider them important for the state of Bavaria. However, we suggest a more extensive follow-up study including a representative sample of social workers from all age groups and from other regions in Germany to validate and substantiate the results.

## Conclusion

FGM/C is an emotional and controversial issue causing often irreparable harm to girls and women. Social workers play an essential role in providing comprehensive care to women and girls affected or endangered by FGM/C. To improve the skills and capacities of social workers to support and accompany women and girls in the best possible way, the following strategies are suggested: (1) enhancing FGM/C knowledge and skills by improving training and information material for the target group, (2) improving referral pathways and addressing deficits in the existing care system, and (3) developing trusting relationships with cultural mediators to build strong community networks.

## Data Availability

The survey and datasets generated and/or analysed during the current study are not publicly available but are available from the corresponding author on reasonable request.

## Footnotes

1 EIGE. *Data Collection on Female Genital Mutilation in the EU*. ISBN: 978-92-9486-067-5.doi: 10.2839/42973. https://eige.europa.eu/sites/default/files/data_collection_on_fgm_in_the_eu.pdf.

2 In Bavaria very few independent FGM/C advice centers (< 5) exist in metropolitan areas. Some pregnancy counselling centres have developed in the last 4 years with funding from the Bavarian State Ministry of Social Affairs a specific expertise to offer advice on FGM/C in Bavaria.
